# Sequential platinum and PARP Inhibition enhances PD1 immunotherapy efficacy in murine *Brca2* mutated pancreatic cancer

**DOI:** 10.1038/s41598-026-35423-7

**Published:** 2026-01-31

**Authors:** John C. McVey, Max M. Wattenberg, Heather Coho, Kayjana Infante, Kelly Markowitz, Devora Delman, Cynthia Clendenin, Emma E. Furth, Rashmi Tondon, Ben Z. Stanger, Robert H. Vonderheide, Kim A. Reiss, Gregory L. Beatty

**Affiliations:** 1 Abramson Cancer Center, Perelman School of Medicine, University of Pennsylvania, Philadelphia, PA USA; 2 Department of Surgery, Perelman School of Medicine, University of Pennsylvania, Philadelphia, PA USA; 3 Division of Hematology-Oncology, Department of Medicine, Perelman School of Medicine, University of Pennsylvania, Philadelphia, PA USA; 4 Department of Medicine, Washington University School of Medicine in St. Louis, St. Louis, MO USA; 5 Department of Pathology, Abramson Cancer Center, University of Pennsylvania, Philadelphia, PA USA; 6 Perelman School of Medicine, University of Pennsylvania, 3400 Civic Center Blvd, South Pavilion, Rm 8-107, Philadelphia, PA 19104 USA

**Keywords:** Cancer, Immunology

## Abstract

Pancreatic ductal adenocarcinoma (PDAC) is a challenging malignancy to treat, but emerging evidence suggests that specific subtypes may respond more favorably to certain therapies. *BRCA*-mutated PDAC represents a distinct subtype that is particularly sensitive to DNA-damaging therapies. The current standard of care for advanced *BRCA*-mutated PDAC involves induction platinum-based chemotherapy followed by maintenance therapy with a poly (ADP-ribose) polymerase inhibitor (PARPi). However, the randomized phase III POLO trial, upon which this standard is based, did not demonstrate an improved overall survival in patients who received olaparib compared to those who received placebo, highlighting the need for new therapeutic approaches. Additionally, there is a lack of robust models that recapitulate the tumor microenvironment of *BRCA* mutated PDAC, limiting the development of next-generation maintenance treatment options. In this study, we developed a syngeneic and immunocompetent mouse model of *Brca2*-mutated PDAC. The model demonstrated high sensitivity to cisplatin plus gemcitabine, but limited efficacy of PARPi monotherapy. Induction with platinum-based chemotherapy sensitized tumors to PARPi maintenance therapy and promoted an exhausted, T cell-inflamed tumor microenvironment. However, resistance emerged which was associated with CDX2 expression and tumor differentiation. The addition of anti-PD1 treatment to PARPi maintenance enhanced tumor regression and prolonged overall survival. These findings provide preclinical support for ongoing clinical trials investigating immunotherapy with PARPi as a maintenance strategy in homologous recombination-deficient PDAC.

**Supplementary Information:**

The online version contains supplementary material available at 10.1038/s41598-026-35423-7.

## Introduction

Pancreatic ductal adenocarcinoma (PDAC) is the third leading cause of cancer-related death with a dismal 5-year overall survival rate of just 8%^1^. Emerging evidence highlights the importance of genetically distinct PDAC subtypes, which have been shown to respond to targeted therapies. Notably, tumors with deficiencies in homologous recombination (HRD), a critical DNA repair pathway, exhibit heightened sensitivity to DNA-damaging therapies^2–6^. In PDAC, HRD is frequently driven by loss-of-function mutations in the *BRCA* gene, which can be observed in approximately 3–8% of patients^7,8^.

The current therapeutic approach for *BRCA*-related PDAC includes first-line treatment with platinum-based chemotherapy for four to six months, followed by maintenance treatment with a poly (ADP-ribose) polymerase inhibition (PARPi). However, in the phase III POLO trial of maintenance olaparib versus placebo in patients with germline *BRCA* mutations and metastatic PDAC, the median progression free survival in the treatment group was a mere 7.4 months and overall survival was no different between the arms^9^. This modest result highlights the need for next-generation treatment strategies for this patient population. Yet, a lack of robust model systems to study *BRCA*-related PDAC has limited the development of next-generation maintenance treatment options.

Reductive models, such as patient derived organoids and xenograft models, have been extensively used to study *BRCA*-related PDAC^10,11^. One limitation of these approaches is a lack of inclusion of tumor microenvironment (TME) elements, such as the immune system, that may play an important role in defining response to therapy^12^. Indeed, immunosuppressive macrophages and T cells have been suggested to regulate the response to PARPi in *BRCA*-related breast and ovarian cancers^13^. As the PDAC TME is characterized by dense fibrosis, immunosuppressive myeloid cell infiltration and low T cell infiltration^14^, modeling the TME in *BRCA*-related PDAC may be especially critical to identify new clinically relevant treatment strategies.

In this study, we developed a syngeneic and immunocompetent mouse model of *Brca2* mutated PDAC. In doing so, we found that loss of BRCA2 function maintains an immunologically cold and T cell excluded tumor microenvironment. Additionally, the *Brca2*-mutated PDAC mouse model replicated treatment findings observed in human *BRCA* mutant PDAC such as increased sensitivity to DNA damaging chemotherapy followed by limited durability of maintenance PARPi monotherapy. Using this model, we found that induction platinum-based chemotherapy induced an exhausted T cell inflamed tumor microenvironment which was effectively targeted by combining anti-PD1 with PARPi therapy.

### Methods

*Human samples.* PDAC resection specimens from patients with germline mutation in *BRCA2* (Supplementary Table S1) were collected at the University of Pennsylvania under a protocol approved by the University of Pennsylvania Institutional Review Board. Written informed consent was obtained from all patients and studies were performed in accordance with the Declaration of Helsinki. All human studies were conducted in accordance with the relevant guidelines and regulations.

*Mice.* Eight- to twelve-week-old male and female C57BL/6 (stock. No. 000664) mice were bred in-house or ordered from Jackson Laboratory. *LSL-Kras*^*G12D/+*^ *LSL-Trp53*^*R172H/+*^ *Brca2*^*exon11f/f*^ *Pdx-1*-Cre *YFP* (KPCB-Y) mice were generated and bred in the Pancreatic Cancer Mouse Hospital of the Abramson Cancer Center at the University of Pennsylvania. KPCB-Y mice were congenic with C57BL/6 mice based on DartMouse Genetic Background Check, and genetics were determined by Transnetyx genotyping. However, genomic analysis at the Mouse Congenic Core Facility at Dartmouth University revealed incomplete BRCA2 exon 11 recombination, likely due to transient PDX-1 Cre activity in vivo, resulting in partial deletions in the spontaneous tumor model. Consequently, this technical challenge necessitated derivation of clonal cell lines via single-cell sorting of YFP^+^ tumor cells (see *Cell lines* section). Animal housing was under pathogen-free conditions in a barrier facility. The Institute of Animal Care and Use Committee at the University of Pennsylvania reviewed and approved all animal protocols. This study is reported in accordance with ARRIVE guidelines. All animal studies were conducted in accordance with the relevant guidelines and regulations.

*Cell lines.* Polyclonal PDA.69 and monoclonal 6694c2 *Brca2* wild type cell lines were derived from KPC mice as previously described^15,16^. For derivation of *Brca2* null PDA cell lines, *Brca2*^*exon11f/f*^ mice were initially crossed with *Pdx-1 -Cre; Rosa26* ^*YFP/YFP*^ mice. The resultant offspring (CB-Y) were then crossed with *Kras*^*LSL−G12D/+*^; *Trp53*^*LSL − R172H/LSL−R172H*^ (KP) mice to generate KPCB-Y mice. This spontaneous model of BRCA2-deficient pancreatic adenocarcinoma was technically challenging to maintain due to the complexity of five separate alleles but enabled tumor development in the context of an immunocompetent host under physiological selection pressures. A pancreatic tail tumor from a female KPCB-Y mouse was processed as previously described^17^. YFP-expressing cells were isolated by single-cell sorting into 96 well tissue culture plates (Celltreat) using a FACS Aria II (BD). Clonal populations were expanded, and two distinct clones were selected for further study: 23223T-18 (designated KPCB.c1) and 23223T-19 (designated KPCB.c2). *Brca2* exon 11 deletion was confirmed using PCR. These two clonal lines represent distinct subpopulations within the original heterogeneous tumor. Cell culture was performed using DMEM (Corning) with 10% FBS (VWR), 1% L-glutamine (Thermo Fisher Scientific) and 83 µg/mL gentamicin (Thermo Fisher Scientific). Human ASPC1 and CAPAN1 cells were obtained from ATCC. ASPC1 were cultured using RPMI (Corning) with 10% FBS (VWR), 1% L-glutamine (Thermo Fisher Scientific) and 83 µg/mL gentamicin (Thermo Fisher Scientific). CAPAN1 cells were cultured using IMDM (Gibco) with 20% FBS (VWR), 1% L-glutamine (Thermo Fisher Scientific) and 83 µg/mL gentamicin (Thermo Fisher Scientific). All cells were cultured at 37 °C and 5% CO2. All cell lines used tested negative for mycoplasma contamination.

*DNA isolation and PCR.* Genomic DNA was extracted from clonal KPC BRCA2 wild type and KPCB cell lines as per the manufacturers protocol using a QIAamp DNA minikit (Qiagen). PCR was performed to detect *Brca2* using primers FWD: 5’ GATTTACCTGCCGATCAAGG and REV: 5’ CTGTCCTTCAGGGGTTTTGA on a MiniAmp Thermal Cycler (Thermo Fisher Scientific). The PCR primers used detected regions within exon 11 of the *Brca2* gene that generated a 629 bp PCR product. This approach was used since exon 11 is greater than 3,500 bp thus limiting efficient amplification by standard PCR.

*Animal treatment protocol.* For in vivo tumor models, a single injection of 1 × 10^6^ cells were implanted subcutaneously into the flank of each mouse. Sex-matched eight- to twelve-week-old mice were block randomized in an unblinded manner. Sample sizes were estimated based on pilot studies to provide sufficient numbers of mice in each treatment group for statistical analysis. Sample sizes ranged from 3 to 16 mice per group depending on the experiment. Mice were monitored three times per week and euthanized using previously defined criteria including tumor volume > 1000 mm^3^, loss of > 20% body weight, or signs of distress. Euthanasia was performed by CO₂ asphyxiation followed by cervical dislocation, in accordance with the University of Pennsylvania IACUC guidelines. Gemcitabine (120 mg/kg) suspended in PBS and cisplatin (4 mg/kg) suspended in 0.9% sodium chloride were administered by intra-peritoneal (i.p.) injection weekly for one to four doses depending on the experiment. Olaparib (50 mg/kg) suspended in 4% DMSO, 30% PEG300 and 66% ddH2O was administered daily by i.p. injection until experiment endpoint. Anti-PD1 (clone RMP1-14, Catalog #: BE0146, RRID: AB_10949053, 0.2 mg) and anti-CTLA-4 (clone 9H10, Catalog #: BE0131, RRID: AB_10950184, 0.2 mg) antibodies were suspended in 200 µL PBS and administered i.p. every 3–4 days for four doses to mice via a 30-gauge needle and after sterilization of the abdomen.

*In vitro cell survival assay.* Cells were seeded at a density of 5000 per well in 96-well tissue culture plates (Celltreat). After 24 h, the media was replaced with media containing either cisplatin or olaparib at the indicated concentrations. Cell viability was measured at 48 h (cisplatin) or 96 h (olaparib) using the MTT assay (Promega) according to the manufacturer’s protocol.

*Formalin-fixed paraffin embedded cell pellets.* Cells were pelleted into 200 µL PCR tubes by centrifugation at 1500 RPM for 5 min at room temperature. Cell pellets were fixed in 10% formalin (Sigma) for 24 h at room temperature and subsequently embedded in HistoGel (VWR) at 4 °C for 1 h. HistoGel embedded cell pellets were stored in 70% ethanol at 4 °C before paraffin embedding.

*Flow cytometry.* Antibody staining and acquisition of flow cytometry was conducted as previously described^17^. In brief, tumors were resected and minced into 1 mm pieces using micro-dissecting scissors on ice in DMEM containing collagenase (1 mg/mL, Sigma-Aldrich) and DNase (150 U/mL, Sigma). Tissues were then incubated at 37 °C for 45 min with intermittent shaking. Tissue suspensions were filtered through a 70-µm nylon strainer (Corning) before being incubated in ACK lysing buffer (Life Technologies) for 5 min, washed with FACS buffer (PBS, 2% FBS and 2 mM EDTA) and counted using a TC20 automated cell counter (Bio-Rad). Single cell suspensions were stained with Aqua dead cell stain kit (Life Technologies) for 20 min at room temperature followed by a master mix of primary conjugated antibodies (Supplementary Table S2) for 30 min at 4 °C. Flow cytometry data was acquired on a FACSCanto II (BD Biosciences) and analysis performed using FlowJo version 10.2 (BD).

*Microscopic analysis.* Tissues were processed and embedded as previously described^18^ to produce formalin-fixed paraffin embedded (FFPE) blocks. 5 μm sections were obtained from each sample and placed onto Superfrost Plus microscope slides (VWR International). The Ventana Discovery Ultra automated slide staining system (Roche) was used for automated immunohistochemistry staining of tissues. For mouse antigens, the following primary antibodies were used: Ki67 (Cell Signaling, Clone D3B5, Catalog #: 12202, RRID: AB_2620142) and CK19 (Abcam, Clone EPNCIR127B, Catalog #: ab133496, RRID: AB_11155282). For human antigens, the following primary antibodies were used: Ki67 (Roche, Clone 30 − 9, Catalog #: 790–4286, RRID: AB_2631262) and CK19 (Roche, Clone A53-B/A2.26, Catalog #: 760–4281, RRID: AB_2335655). CD3 (Roche, Clone 2GV6, Catalog #: 790–4341, RRID: AB_2335978) and Phospho-histone H2A.X (Cell Signaling, Clone 20E3, Catalog #: 9718, RRID: AB_2118009) was used for both mouse and human antigens. Hematoxylin (Roche), bluing reagent (Roche) and purple, DAB, yellow and teal chromogens (Roche) were used.

*Immunohistochemistry image analysis.* An Aperio C2 scanner (Leica) was used to obtain whole slide images. For quantification of phospho-histone H2A.X in mouse tumor cell pellets, phospho-histone H2A.X positive cells were manually counted in four representative 20x fields per sample in a blinded manner. For all other analyses, Visiopharm Integrator System software (Version 2020.01) was used for detection and classification of cells based on colorimetric features within manually defined regions of interest (ROI). Cell numbers were normalized to ROI area and reported as cells per mm^2^ or as % of ROI.

*RNA isolation and sequencing.* RNA isolation and sequencing was performed as previously described^18^. Briefly, total RNA was extracted using the RNeasy FFPE kit (Qiagen) from multiple 5 μm serial sections cut from FFPE tissues were submitted to Wistar Institute for Genomics facility for library preparation, quantitation and single read, 100 bp Next Generation Sequencing on a NextSeq 2000 (Illumina).

*Analysis of RNA-seq data.* The BaseSpace Suite (Illumina) was used to map reads to the GRCm38/mm10 genome. DESeq2 (v1.32.0)^19^ was used for gene expression normalization and differential gene expression (DEG) analysis across groups. Volcano plots and PCA using all detected genes with regular log transformation were performed in DESeq2. Gene set enrichment analysis was performed using GSEABase (v1.54.0) in R. RNAseq data have been deposited at the Gene Expression Omnibus (GEO) under accession numbers GSE300887 https://www.ncbi.nlm.nih.gov/geo/query/acc.cgi? acc=GSE300887) and GSE300888 (https://www.ncbi.nlm.nih.gov/geo/query/acc.cgi? acc=GSE300888).

*Statistical analysis.* Mann-Whitney *U* tests were used for comparison of unpaired variables with a two-sided alpha of 0.05. For multiple comparisons, one- or two-way ANOVA with Tukey’s test with an FDR < 0.05 was used. To compare in vitro response curves to cisplatin or olaparib in our cell lines, two-way ANOVA was used to test the null hypothesis that the treatment response in the control cell line vs. *Brca2* mutated cell line was indistinguishable. Overall survival analyses were performed using Kaplan-Meier methodology and the log-rank test (Mantle Cox) was used to test for significance between groups. Statistical significance is denoted as *, *p* < 0.05; **, *p* < 0.01; ***, *p* < 0.001 and ****, *p* < 0.0001. Non-significant results are not indicated on the figures. Statistical testing was performed in Prism (GraphPad Software, version 9.2.0).

### Results

**Fig. 1:**
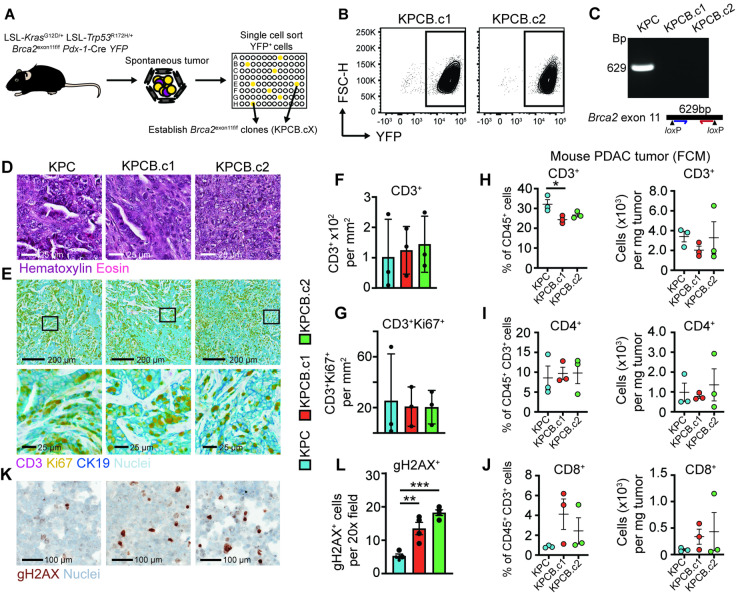
A murine model of *Brca2* mutant pancreatic cancer. **(A)** Schematic describing generation of *Brca2* mutated pancreatic cancer cell line. **(B)** Flow cytometry dot plot showing YFP^+^ expression in tumor cell clones. **(C)** PCR showing loss of exon 11 in clonal pancreatic cancer cell lines. **(D)** Representative H&E images of implanted tumors from well differentiated (KPCB.c1) and poorly differentiated (KPCB.c2) clones. Scale bar: 25 μm. **(E)** Immunohistochemistry analysis of T cell infiltration into implanted tumors. Black box represents insert. Scale bar: 200–25 μm. **(F)** Quantification of T cells from panel E (*n* = 3 per group). **(G)** Quantification of proliferating T cells from panel E (*n* = 3 per group). **(H)** Flow cytometry analysis of T cells from implanted tumors (*n* = 3 per group). **(I)** Flow cytometry analysis of CD4^+^ T cells from implanted tumors (*n* = 3 per group). **(J)** Flow cytometry analysis of CD8^+^ T cells from implanted tumors (*n* = 3 per group). **K)** Immunohistochemistry for double stranded DNA breaks (gH2Ax) in pelleted KPC, KPCB.c1 and KPCB.c2 cell lines. **L)** Quantification of panel K (*n* = 4 per group). One way ANOVA with Tukey correction for multiple comparisons was used to determine statistical significance. Statistical significance denoted as *, *p* < 0.05; **, *p* < 0.01; ***, *p* < 0.001 and ****, *p* < 0.0001.

*Loss of BRCA2 function is insufficient to alter T cell exclusion in the PDAC tumor microenvironment.* We first generated clonal tumor cell lines (termed KPCB) from spontaneous tumors arising in a genetically engineered mouse model of *Kras*^*G12D*^, *Trp53*^*R172H*^ and *Brca2* exon 11 deleted PDAC which were congenic with C57BL/6 mice (Fig. 1A). Two selected clones (KPCB.c1 and KPCB.c2) exhibited expression of the yellow fluorescent protein (YFP) epithelial lineage marker and loss of *Brca2* exon 11, confirming their genetic identity (Fig. 1B-C; Supplemental Fig. 1). After subcutaneous implantation into congenic C57BL/6 mice, KPCB clones developed tumors ranging from well-differentiated (KPCB.c1) to poorly differentiated (KPCB.c2) adenocarcinoma (Fig. 1D). Immunohistochemistry (IHC) analysis revealed that *Brca2* loss did not significantly alter CD3^+^ T cell infiltration, or CD3^+^ T cell proliferation compared to *Brca2* wild-type tumors (Fig. 1E-G). Additionally, flow cytometry analysis showed no substantial differences in T cell or myeloid cell populations based on *Brca2* status (Fig. 1H-J; Supplemental Fig. 2A-B). However, *Brca2* mutated tumors exhibited an increase in γH2AX^+^ tumor cells, indicating elevated spontaneous DNA damage compared to wild-type tumors (Fig. 1K-L). These findings suggest that HRD alone does not necessarily induce significant changes in the immune TME of PDAC.

**Fig. 2:**
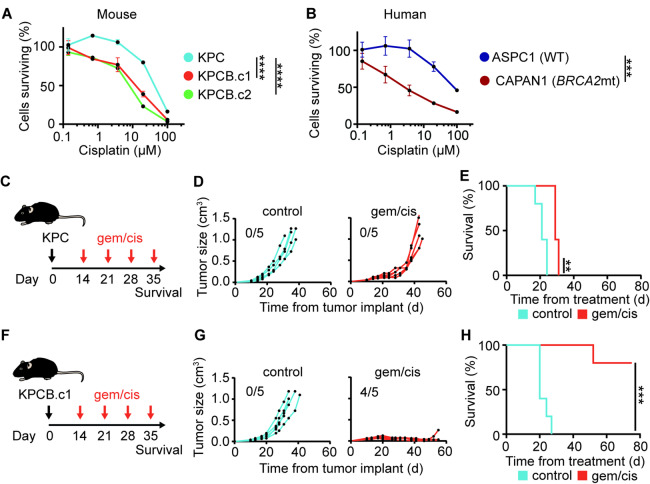
BRCA2 mutated pancreatic cancer is sensitive to platinum-based chemotherapy. **(A)** MTT assay of KPC, KPCB.c1 or KPCB.c2 at varying doses of cisplatin (*n* = 3 per group). **(B)** MTT assay of ASPC1 (human PDAC non-BRCA mutated) and CAPAN1 (human PDAC BRCA2 mutated) cell lines at varying doses of cisplatin (*n* = 3 per group). **(C)** Experimental design for D-E (*n* = 5 per group). **(D)** Tumor growth curves. Numbers represent survival at end of experiment. **(E)** Kaplan-Meier plot. **(F)** Experimental design for G-H (*n* = 5 per group). **(G)** Tumor growth curves. Numbers represent survival at end of experiment. **(H)** Kaplan-Meier plot. Two way ANOVA was used to compare MTT assays between KPC and KPCB.c1 or KPCB.c2 treated with cisplatin. For the rest of the data, one way ANOVA with Tukey correction for multiple comparisons and Log-rank test were used to determine statistical significance. Statistical significance denoted as n.s. *p* > 0.05; *, *p* < 0.05; **, *p* < 0.01; ***, *p* < 0.001 and ****, *p* < 0.0001.

*Murine BRCA2 mutated PDAC is sensitive to platinum-based chemotherapy*,* mirroring treatment responses observed in humans.* In human PDAC, DNA repair deficiencies enhance susceptibility to DNA-damaging therapies such as platinum-based chemotherapy^6^. To determine if this held true in our model, we treated *Brca2* mutated KPCB clones in vitro with increasing doses of cisplatin and compared them to *Brca2* wild-type KPC cells. *Brca2* mutated cells exhibited greater sensitivity, with significantly fewer surviving cells, a finding also observed in human *BRCA2* mutated CAPAN1 cells compared to *BRCA2* wild-type ASPC1 cells (Fig. 2A-B). We next evaluated treatment responses in vivo using a subcutaneous mouse model (Fig. 2C, F). Mice bearing *Brca2* wild-type KPC tumors showed minimal tumor control and survival benefit with gemcitabine/cisplatin treatment (Fig. 2D-E). In contrast, KPCB.c1 tumors were highly sensitive, with 4 of 5 mice achieving complete tumor regression (Fig. 2G-H). These findings confirm that the *Brca2* mutated model recapitulates the platinum-based chemotherapy sensitivity observed in human *BRCA2* mutated PDAC.

*PARP inhibitor monotherapy has some in vivo efficacy against murine Brca2 mutated PDAC.* While KPCB.c1, KPCB.c2, and CAPAN1 cells showed increased sensitivity to olaparib in vitro (Supplemental Fig. 3A-B), olaparib treatment in vivo showed limited tumor growth control (Supplemental Fig. 3C-D). Specifically, three of five mice did not respond to olaparib monotherapy while two of five mice showed modest tumor growth control, however, there was no survival benefit between controls and olaparib monotherapy treated mice (Supplemental Fig. 3E). Flow cytometry analysis of tumors treated daily with PARPi for 10 days showed that there were no substantial changes in immune cell or T cell infiltration into the TME (Supplemental Fig. 3 F and G). Additionally, bulk RNA sequencing analysis showed that PARPi treated tumors did not differ significantly from control tumors in DNA damage response genes (911 genes from “Gene Orthology: DNA Damage Response”) (Supplemental Fig. 3H and I). Finally, IHC analysis showed that γH2AX^+^ cells were slightly increased from 0.93% to 1.71% in control vs. PARPi treated tumors (Supplemental Fig. 3 J and K). These findings demonstrate that PARPi monotherapy exhibits mild therapeutic efficacy in vivo against *Brca2*-mutated murine PDAC.

**Fig. 3:**
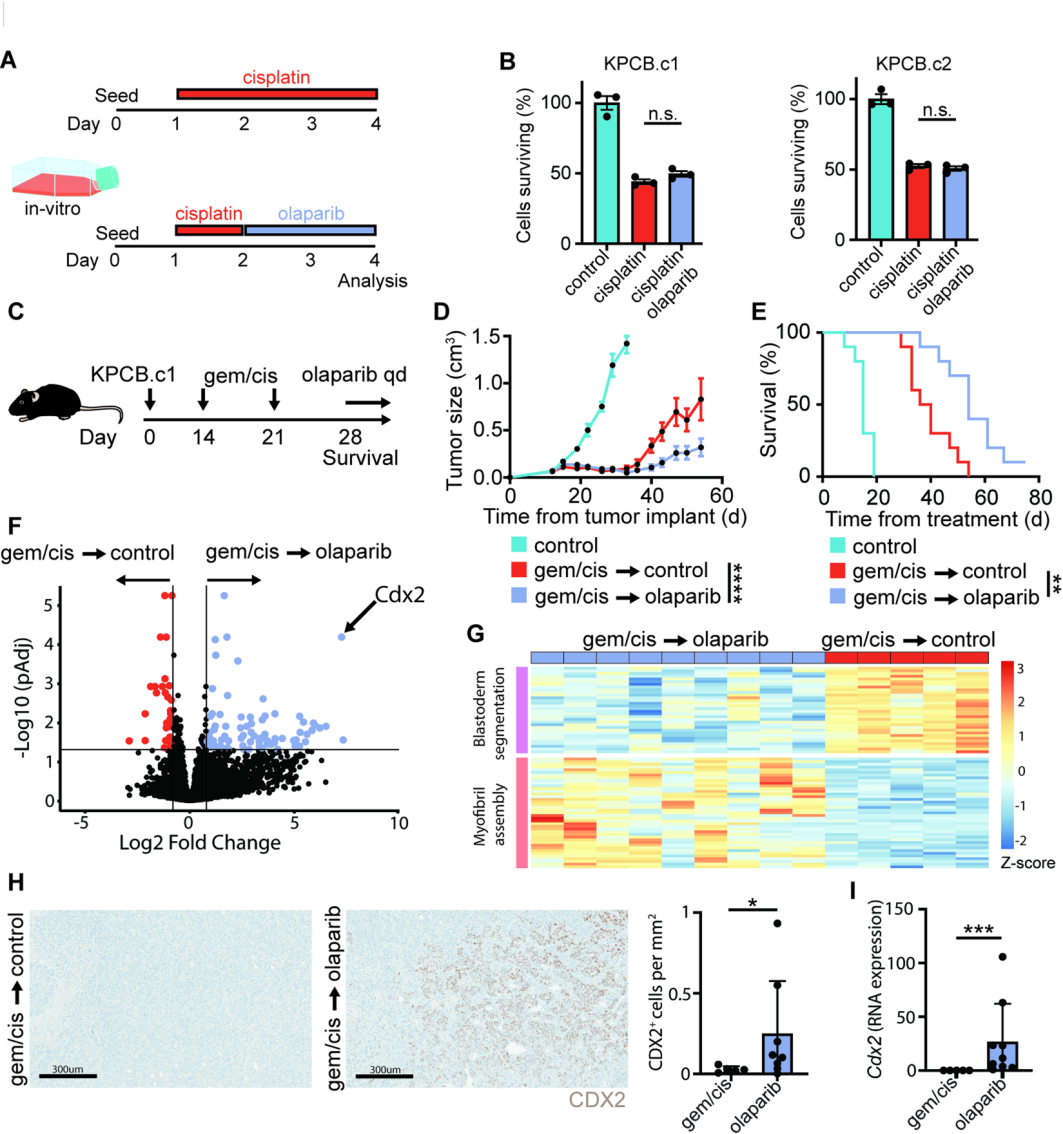
PARP inhibition maintenance therapy improves survival, but resistance develops in a *Brca2* mutated pancreatic cancer model. **(A)** Experimental design for B (*n* = 3 per group). **(B)** MTT assay of KPCB.c1 or KPCB.c2 after treatment with cisplatin or cisplatin followed by olaparib. **(C)** Experimental design for D-E (*n* = 10 per group). **(D)** Tumor growth curves. **(E)** Kaplan-Meier plot. **(F)** Volcano plot showing differentially expressed genes in tumors at the time of death between gemcitabine/cisplatin only treated and PARPi maintenance treated mice. *Cdx2* is labeled as the highest differentially expressed gene (*n* = 5 in control maintenance and *n* = 9 in olaparib maintenance). **(G)** Heatmap of gene set enrichment analysis results from differentially expressed genes in F. **(H)** Representative and quantification of CDX2 staining from tumors at the time of death in mice treated with gemcitabine/cisplatin only or PARPi maintenance (*n* = 5 in control maintenance and *n* = 9 in olaparib maintenance). **(I)** *Cdx2* expression from bulk RNA sequencing shown in F (*n* = 5 in control maintenance and *n* = 9 in olaparib maintenance). Mann-Whitney test, one way ANOVA with Tukey correction for multiple comparisons and Log-rank test were used to determine statical significance. Statistical significance denoted as n.s. *p* > 0.05; *, *p* < 0.05; **, *p* < 0.01; ***, *p* < 0.001 and ****, *p* < 0.0001.

*Resistance to maintenance olaparib following induction chemotherapy occurs in a Brca mutated mouse model.* Current treatment for *BRCA*-mutated PDAC involves first-line platinum-based chemotherapy, such as gemcitabine and cisplatin, for 4–6 months followed by PARPi maintenance^9^. As PARPi monotherapy showed minimal activity in mouse PDAC models, but maintenance therapy is clinically effective, we hypothesized that platinum-based chemotherapy sensitizes tumor cells to PARPi-mediated killing. In vitro experiments showed no additive or synergistic cell killing when PARPi was administered after cisplatin treatment (Fig. 3A-B). In contrast, in mice bearing subcutaneous KPCB.c1 tumors, induction chemotherapy, with the clinically relevant gemcitabine/cisplatin, followed by PARPi maintenance led to improved tumor response and prolonged survival compared to no maintenance therapy (Fig. 3C-E). However, PARPi maintenance did not trigger tumor cures. Bulk RNA sequencing analysis of tumors at the time of death showed several differentially expressed genes (DEGs) between no maintenance and PARPi maintenance treated mice (Fig. 3F). Olaparib treated tumors showed downregulation of “Blastoderm Segmentation” and upregulation of “Myofibril Assembly” associated genes, compared to those treated with platinum-based induction chemotherapy without maintenance therapy (Fig. 3G). Additionally, the embryologic transcription factor *Cdx2* was the highest differentially expressed gene in olaparib treated tumors as compared to no maintenance treated tumors (Fig. 3F). IHC analysis confirmed expression of CDX2 in all olaparib treated tumors, while minimal expression was seen in no treatment tumors (Fig. 3H-I). These findings suggest that platinum-based chemotherapy sensitizes *Brca2* mutant tumors to PARPi by modifying the tumor or host microenvironment and show that resistance is associated with CDX2 expression and tumor differentiation.

**Fig. 4:**
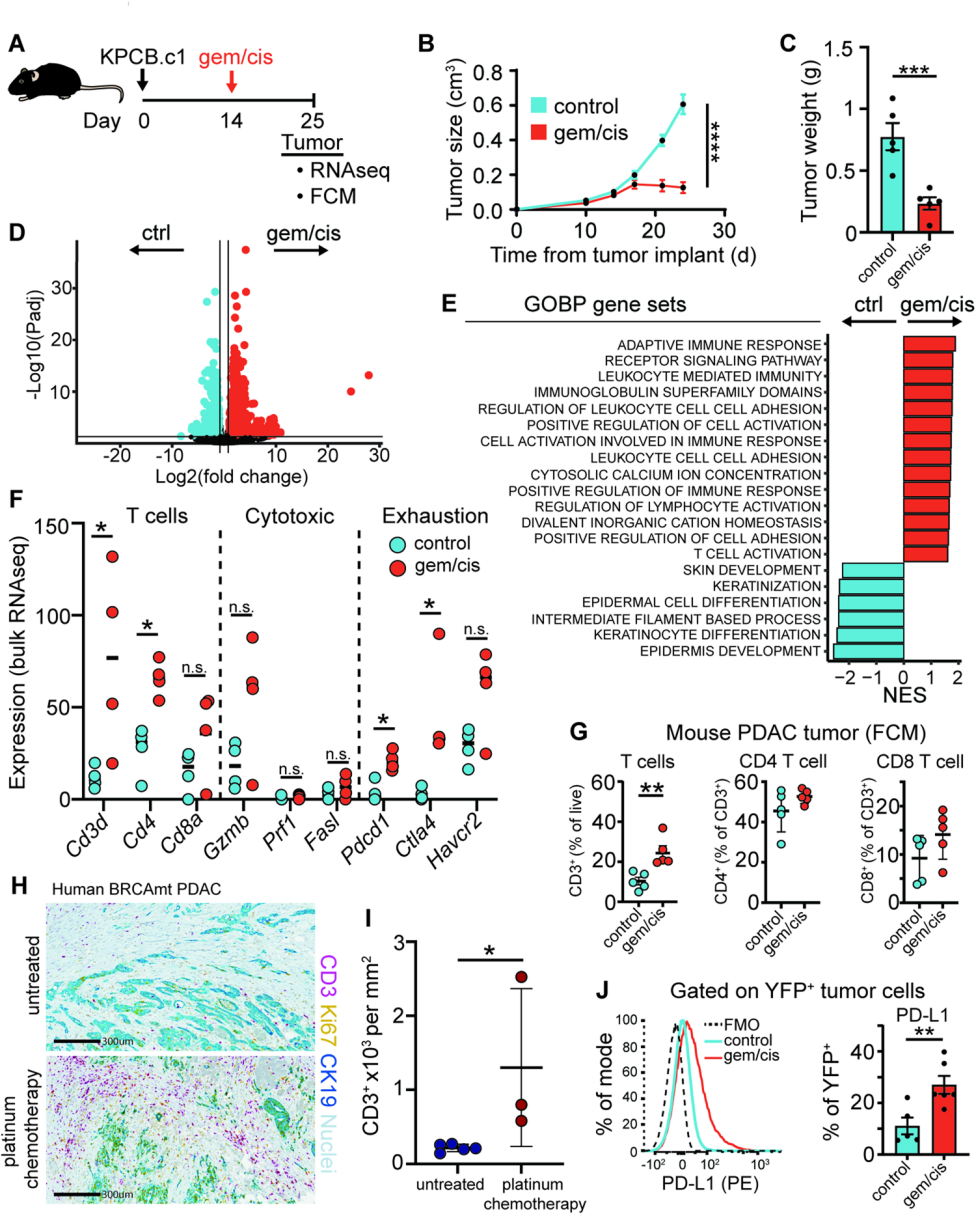
Induction chemotherapy induces an inflamed T cell exhausted tumor microenvironment. **(A)** Experimental design for B-G (*n* = 5 per group). **(B)** Tumor growth curves. **(C)** Tumor weights at day 25. **(D)** Volcano plot of differentially expressed genes from bulk RNA sequencing of control or gemcitabine/cisplatin treated mice (*n* = 4 per group). **(E)** Gene ontology and biological process pathway analysis of differentially expressed genes from panel D. **(F)** Gene expression of T cell and T cell phenotype markers from bulk RNA sequencing (*n* = 4 per group). **(G)** Flow cytometry analysis of T cell, CD4^+^ and CD8^+^ T cell infiltration from untreated and gemcitabine/cisplatin treated BRCA2 mutated tumors (*n* = 5 per group). **(H)** Representative immunohistochemistry images from human *BRCA-* or *PALB2*-mutated pancreatic cancer surgical resection specimens that received neoadjuvant platinum-based chemotherapy or upfront surgery. One patient in the neoadjuvant treatment group was excluded using Grubbs outlier test (*n* = 5 in up front surgery and *n* = 3 in neoadjuvant chemotherapy group). **(I)** Quantification of CD3 positive cells from human immunohistochemistry samples. **(J)** Flow cytometry analysis of PD-L1 expression on untreated and gemcitabine/cisplatin treated *Brca2* mutated tumors (*n* = 5 per group). One way ANOVA with Tukey correction for multiple comparisons or Mann-Whitney test was used to determine statical significance. Statistical significance denoted as *, *p* < 0.05; **, *p* < 0.01; ***, *p* < 0.001 and ****, *p* < 0.0001. The following abbreviations are used in the figure, FCM, flow cytometry; GOBP, gene ontology biological process; NES, normalized enrichment score; and FMO, florescence minus one.

*Induction chemotherapy induces a T cell-inflamed but exhausted tumor microenvironment.* We next investigated the mechanisms by which platinum-based chemotherapy might condition tumors for maintenance therapy, aiming to provide a rationale for novel maintenance strategies. Tumors were harvested ten days post-gemcitabine/cisplatin treatment (Fig. 4A). Treatment induced an initial tumor response, although tumors persisted (Fig. 4B-C). Bulk RNA sequencing identified 1,533 upregulated genes in treated tumors and 951 in untreated controls (Fig. 4D). Gene set enrichment analysis (GSEA) showed increased expression of immune-related pathways, including “adaptive immune response”, “leukocyte mediated immunity”, “positive regulation of immune response”, and “T cell activation” (Fig. 4E). Upregulation of *Cd3d* and *Cd4*, alongside exhaustion markers (*Pdcd1* and *Ctla4*), was observed in chemotherapy-treated tumors, though cytotoxic markers (*Gzmb*,* Prf1*,* Fasl*) remained unchanged (Fig. 4F). Flow cytometry confirmed increased tumor-infiltrating T cells without shifts in CD4/CD8 composition (Fig. 4G). Similarly, human *BRCA* or *PALB2* mutated PDAC tumors exhibited increased CD3^+^ staining post-platinum based neoadjuvant chemotherapy (oxaliplatin or carboplatin with gemcitabine or capecitabine) (Fig. 4H-I and Supplemental Table 1). Peripheral blood analysis from the *Brca2* mutated mouse model showed no significant differences in T cell subsets, except an increase in CD4^+^ and PD1^+^ CD4^+^ T cells (Supplemental Fig. 4A-H), suggesting treatment effects are mostly confined to the TME. Notably, PD-L1 expression was elevated on YFP^+^ cancer cells in chemotherapy-treated tumors (Fig. 4J). These findings suggest that platinum-based chemotherapy elicits an immune response in *BRCA*-mutated PDAC, leading to a T cell-inflamed but exhausted TME.

**Fig. 5:**
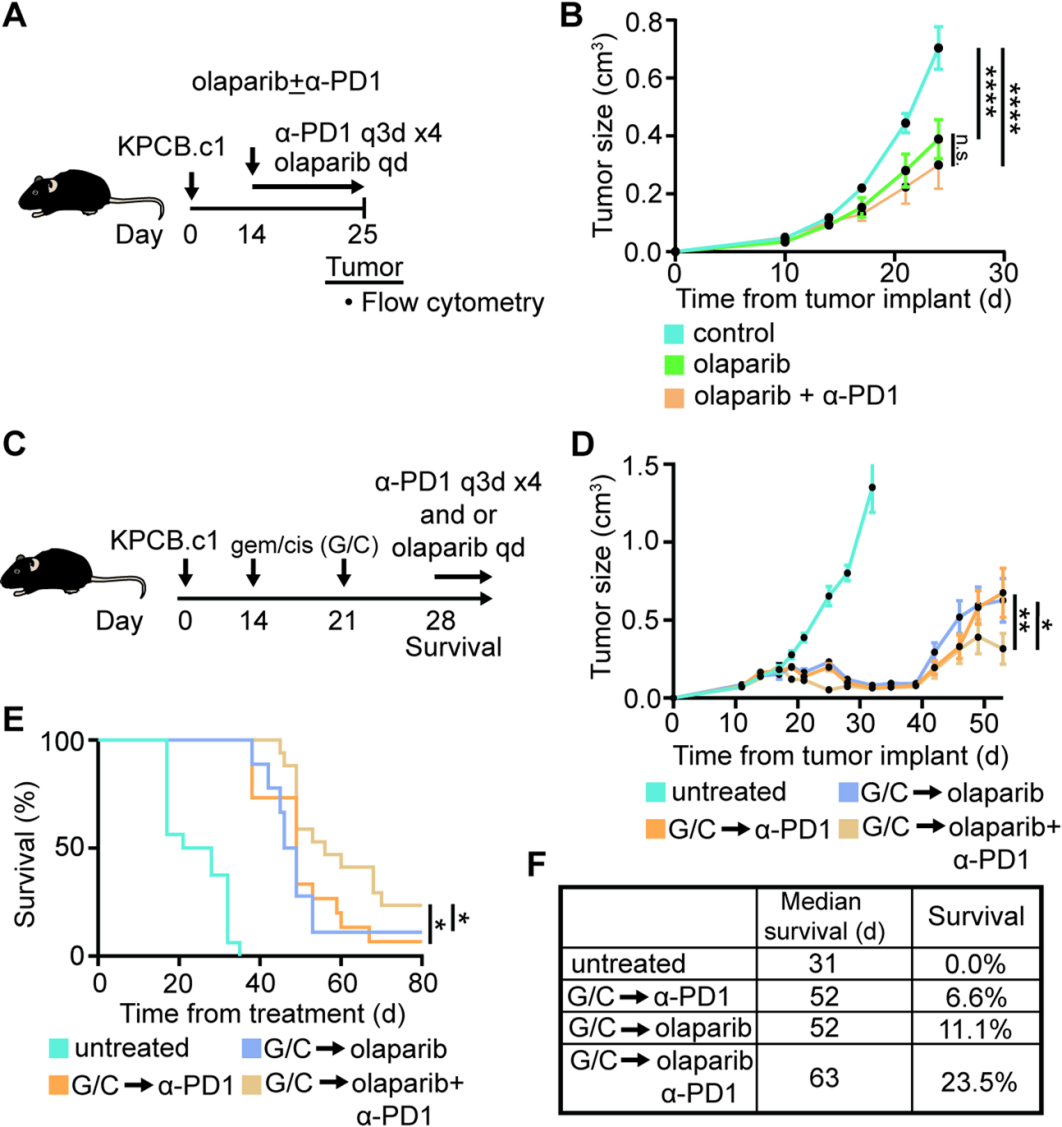
Anti-PD1 therapy improves olaparib maintenance strategy in a *Brca2* mutated mouse model. **(A)** Experimental design for B (*n* = 6 per group). **(B)** Tumor growth curves. **(C)** Experimental design for D-F (*n* = 16 per group). **(D)** Tumor growth curves. **(E)** Kaplan-Meier plot. **(F)** Table showing median survival and percent survival at the end of study. Data are presented up to 95 days post tumor implantation. One way ANOVA with Tukey correction for multiple comparisons and Log-rank test were used to determine statical significance. Statistical significance denoted as n.s. *p* > 0.05; *, *p* < 0.05; **, *p* < 0.01; ***, *p* < 0.001 and ****, *p* < 0.0001.

*Anti-PD1 improves maintenance therapy in murine Brca2 mutated pancreatic cancer.* We next tested the therapeutic activity of immune checkpoint blockade (ICB) in the *Brca2* mutated model. Consistent with the T cell excluded TME observed in this model (Fig. 1), dual ICB therapy (anti-PD1 + anti-CTLA4) failed to control tumor growth in the treatment-naïve setting (Supplemental Fig. 5A-C). Addition of anti-PD1 to PARPi similarly produced no additive benefit over PARPi alone (Fig. 5A-B). Flow cytometry of tumors revealed no changes in T cell or myeloid cell populations within the TME, though a modest increase in tumor-infiltrating CD4 T cells was observed with olaparib plus anti-PD1 compared to untreated controls (Supplemental Fig. 5D-E). Given our observation that platinum-based chemotherapy induces tumor infiltration by T cells with an exhausted phenotype, we hypothesized that immune checkpoint blockade (ICB) might enhance the efficacy of maintenance therapy. To test this, mice bearing *Brca2* mutated tumors received induction gemcitabine/cisplatin followed by maintenance therapy (Fig. 5C and Supplemental Fig. 6 A). Notably, PARPi plus anti-PD1 maintenance therapy led to significantly greater tumor reduction and prolonged survival compared to olaparib or anti-PD1 alone (Fig. 5D-F). However, the addition of anti-CTLA4 to PARPi did not reduce tumor growth or improve overall survival compared to PARPi or anti-CTLA4 alone in the maintenance setting (Supplemental Fig. 6B-D). These findings suggest that anti-PD1 therapy, but not anti-CTLA4 therapy, enhances PARPi maintenance when preceded by platinum-based chemotherapy.

## Discussion

*BRCA2*-related PDAC represents a clinically distinct subtype of PDAC^20,21^. Here, we developed and evaluated an immunocompetent mouse model of *Brca2* mutated PDAC. We demonstrate that the TME of *Brca2* mutated PDAC closely resembles that of wild-type PDAC, characterized by limited T cell infiltration. We further observe that platinum-based chemotherapy sensitizes tumors to maintenance PARPi; however, tumor resistance occurs relatively quickly. Analysis of the TME after induction chemotherapy showed that remodeling occurred and was characterized by a T cell-inflamed yet exhausted phenotype. Finally, we show that adding anti-PD1 therapy to PARPi maintenance treatment enhances therapeutic outcomes in a *Brca2* mutated PDAC mouse model.

The current standard of care for metastatic *BRCA2*-related PDAC involves induction platinum-based chemotherapy followed by maintenance PARPi. While this treatment approach shows significant clinical activity, patients with metastatic *BRCA*-related PDAC still face a median overall survival of approximately two years^6^, a benchmark influenced by the introduction of maintenance olaparib^9^. This underscores the critical need for novel strategies to enhance the efficacy of maintenance therapy. Resistance mechanisms in BRCA-mutant PDAC may involve non-cell-autonomous factors such as stromal reprogramming and an immunosuppressive tumor microenvironment^22,23^. Thus, immunocompetent models of *BRCA*-related PDAC, like the one presented here, provide a platform to dissect these mechanisms and test combinatorial approaches – such as PARPi with immune checkpoint blockade – that target both cancer cell-intrinsic vulnerabilities and tumor microenvironment-mediated resistance.

We found that *BRCA2* mutated PDAC exhibits low baseline T cell infiltration that is comparable to wild-type PDAC, a finding that is consistent with the immune-cold classification of PDAC^14^. However, gemcitabine/cisplatin treatment induced a T cell-inflamed tumor microenvironment characterized by T cell exhaustion. In tumors, such as non-small cell lung cancer and triple-negative breast cancer, cisplatin promotes functional T cell recruitment, potentially through proinflammatory cytokine release that activates type 3 innate lymphoid cells (ILC3) and drives CXCL10-mediated T cell recruitment^24^. In contrast, while neoadjuvant mFOLFIRINOX has been associated with increased T cell exhaustion markers in cancer^25–27^, the immunomodulatory effects of platinum-based chemotherapy in BRCA2-mutated PDAC remain poorly defined. Our study reveals that platinum-based chemotherapy (gemcitabine/cisplatin) primes BRCA2-deficient PDAC for T cell infiltration despite fostering exhaustion. Though the exact mechanism remains unresolved, we hypothesize that the enhanced genomic instability of BRCA2-mutated tumors—rendering them more susceptible to DNA damage—may amplify immunogenic cell death, thereby triggering an adaptive (albeit dysfunctional) immune response.

Our data suggest that platinum-based chemotherapy affects both cancer cells and the TME, ultimately sensitizing tumors to PARPi therapy. In *BRCA*-mutated PDAC, patients exhibit heightened sensitivity to platinum-based chemotherapy^2,6^. However, in a phase II clinical trial, the addition of PARPi to gemcitabine and cisplatin showed no additive benefit, to chemotherapy alone, in *BRCA* and *PALB2* mutated pancreatic cancer^28^. In a single arm phase II trial conducted at our institution, we previously showed that PARPi was effective in the maintenance setting in patients with *BRCA* or *PALB2* mutated PDAC that was sensitive to platinum-based chemotherapy^29^. Additionally, the phase III POLO trial found that maintenance olaparib following induction platinum-based chemotherapy led to improved PFS suggesting that chemotherapy is needed to sensitize tumors to PARPi^9^. Our model recapitulates these clinical findings, showing that olaparib monotherapy in the absence of induction platinum-based chemotherapy has minimal therapeutic impact on *Brca2* mutated tumors in vivo. Bulk RNA sequencing and IHC analyses further revealed that olaparib alone exerts only a modest effect on DNA damage response, despite being the presumed mechanism of action for PARPi in HRD tumors. However, prior treatment with gemcitabine/cisplatin significantly enhanced tumor sensitivity to PARPi, resulting in improved treatment responses that mirror the PFS benefits seen in the POLO trial. It is important to note that our model showed significant sensitivity to gemcitabine and cisplatin chemotherapy (Fig. 2) which raises the possibility that prolonged chemotherapy exposure may improve outcomes in HRD-mutant pancreatic cancer. Indeed, these findings are consistent with clinical data showing response rates as high as 65% in patients with germline BRCA mutant pancreatic cancer treated with gemcitabine/cisplatin^28^. However, chemotherapy intensification is frequently complicated by toxicity in patients and the absence of treatment-limiting toxicities in our mouse model limits direct clinical extrapolation. Therefore, our data suggest that the development of novel DNA damaging therapies that provide the intensity of current combination chemotherapy strategies, while limiting toxicity, may be an opportunity in BRCA mutated pancreatic cancer. Ultimately, although the presented mouse model is an important tool for pre-clinical discovery in BRCA mutant pancreatic cancer, the complex balance of efficacy, toxicity, and resistance to chemotherapy and PARPi in patients must be addressed in future clinical studies. Additionally, future studies are warranted to elucidate the mechanisms by which platinum-based chemotherapy primes tumors for PARPi sensitivity, such as enhanced drug delivery and penetration or induction of T cell anti-tumor immunity.

Despite the observed sensitivity to maintenance PARPi therapy in our model, resistance ultimately developed, with tumors displaying a more differentiated and motile phenotype. Notably, tumors resistant to PARPi maintenance therapy exhibited high expression of the embryologic transcription factor CDX2 compared to tumors that were not treated with maintenance PARPi. Previous studies have suggested that acquired resistance to PARPi in patient-derived xenograft (PDX) models of BRCA1/2-mutated pancreatic cancer is driven by HR restoration^11^. However, reversion events in *BRCA* or *PALB2* mutated pancreatic cancer patients, as assessed by cell free DNA, are rare suggesting that other mechanisms of resistance are at play^30^. Tumors that remain HRD yet fail to respond to therapy are frequently associated with a basal-like molecular subtype^11^. CDX2 has been implicated in the classical subtype of PDAC, which is generally more differentiated than the basal subtype^31^. Our findings suggest that CDX2 may play a role in resistance to maintenance PARPi therapy. However, further mechanistic studies are needed to delineate the precise role of CDX2 in PARPi resistance and to determine whether targeting CDX2-driven pathways could improve therapeutic outcomes in *BRCA*-mutated PDAC. Our data does suggest that CDX2 expression could be used as a biomarker of resistance to PARPi maintenance therapy in *BRCA2* mutated pancreatic cancer.

A recent phase 1b/2 trial showed that the addition of ipilimumab to niraparib in patients with platinum sensitive PDAC was more active than niraparib plus nivolumab in the maintenance setting^32^. This study highlights the potential of combination PARPi and ICB as maintenance therapies for advanced PDAC. Our findings suggest that induction platinum-based chemotherapy, followed by PARPi maintenance, may enhance the sensitivity of *BRCA*-mutated PDAC to anti-PD1 therapy. Notably, both induction chemotherapy and PARPi were required to achieve an improved response to anti-PD1 therapy, suggesting that these treatments may modulate the TME and/or tumor cells in a way that facilitates immune checkpoint blockade efficacy. Despite increased expression of both CTLA-4 and PD-1 in the tumor microenvironment following platinum-based chemotherapy, only PD-1 blockade with PARPi improved tumor control and survival (Fig. 5 and Supplemental Fig. 6). This finding indicates that platinum-based chemotherapy selectively primes the TME in BRCA2-mutant PDAC to be more receptive to anti-PD-1 plus PARPi maintenance therapy rather than anti-CTLA-4 plus PARPi maintenance therapy. The discrepancy of this finding with clinical trial data^32^ showing benefit with anti-CTLA4 plus PARPi in platinum-sensitive patients unselected for BRCA2 mutations may stem from differences in tumor genetics, immune contexture, or patient selection. The combination of PARPi and anti-PD1 is currently being investigated as a maintenance strategy for BRCA-related advanced PDAC in clinical trials (NCT04548752 and NCT04666740) which will be critical in determining whether this combinatorial maintenance approach provides a meaningful clinical benefit for patients with *BRCA*-related PDAC.

A limitation of this study is the reliance on only two *Brca2*-mutated clonal cell lines (KPCB.c1 and KPCB.c2) for in vitro experiments and one (KPCB.c1) for in vivo experiments. Technical challenges, including incomplete BRCA2 exon 11 deletion due to transient PDX-1 Cre activity, low viability post-single-cell sorting (yielding just two expandable clones from one mouse), and failed expansion from a second mouse, limited generation of additional clones. While this may impact the generalizability to other *Brca2*-mutated or heterogeneous PDAC contexts, the consistent chemotherapy/PARPi sensitivity across these clones aligns with clinical platinum-sensitive BRCA2-mutated PDAC, supporting our mechanistic insights.

In summary, we developed an immunocompetent mouse model of *BRCA2*-related PDAC and maintenance PARPi therapy, providing a valuable foundation for the development of next-generation therapeutic strategies for *BRCA*-related PDAC. Using this model, we show that induction gemcitabine/cisplatin induces a T cell inflamed TME that sensitizes tumors to PARPi maintenance therapy. We also show that the addition of anti-PD1 to maintenance therapy strategies may improve outcomes for patients with HRD tumors.

## Supplementary Information

Below is the link to the electronic supplementary material.

Supplementary Material 1

## Data Availability

All data supporting the findings of this study are included in the paper and its Supplementary Information. RNAseq data have been deposited at the Gene Expression Omnibus (GEO) under accession numbers GSE300887 and GSE300888. Additional data are available from the corresponding author upon reasonable request.

## Declarations

### Data transparency

All data supporting the findings of this study are included in the paper and its Supplementary Information. RNAseq data have been deposited at the Gene Expression Omnibus (GEO) under accession numbers GSE300887 and GSE300888. Additional data are available from the corresponding author upon reasonable request.
