# Supplementary material for: Sequential platinum and PARP Inhibition enhances PD1 immunotherapy efficacy in murine Brca2 mutated pancreatic cancer
Source: Sci Rep. 2026 Jan 31;16:6808. doi: 10.1038/s41598-026-35423-7 (PMC12916747; doi:10.1038/s41598-026-35423-7)
Supplement: Supplementary file 1 — Supplementary Material 1 [file 41598_2026_35423_MOESM1_ESM.pdf]

Supplemental Figure 1

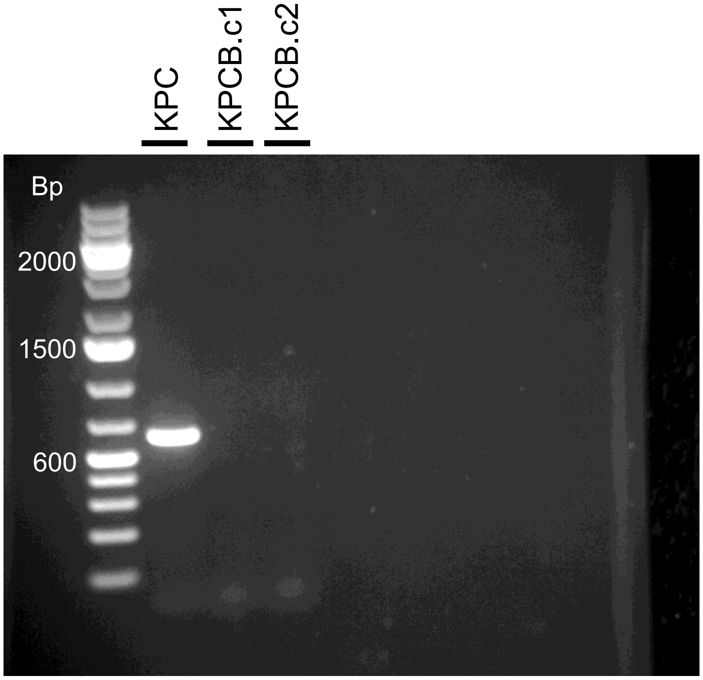

Supplemental Figure 1. PCR analysis for exon 11 deletion in pancreatic cancer cell lines. Full-length PCR blot corresponding to Figure 1C. This image shows the complete membrane with visible edges for the PCR analysis of exon 11 loss in pancreatic cancer clonal cell lines KPC (6694c2), KPCB.c1, and KPCB.c2. No membrane cutting was performed prior to hybridization. The cropped region shown in Figure 1C is indicated for clarity.

Supplemental Figure 2

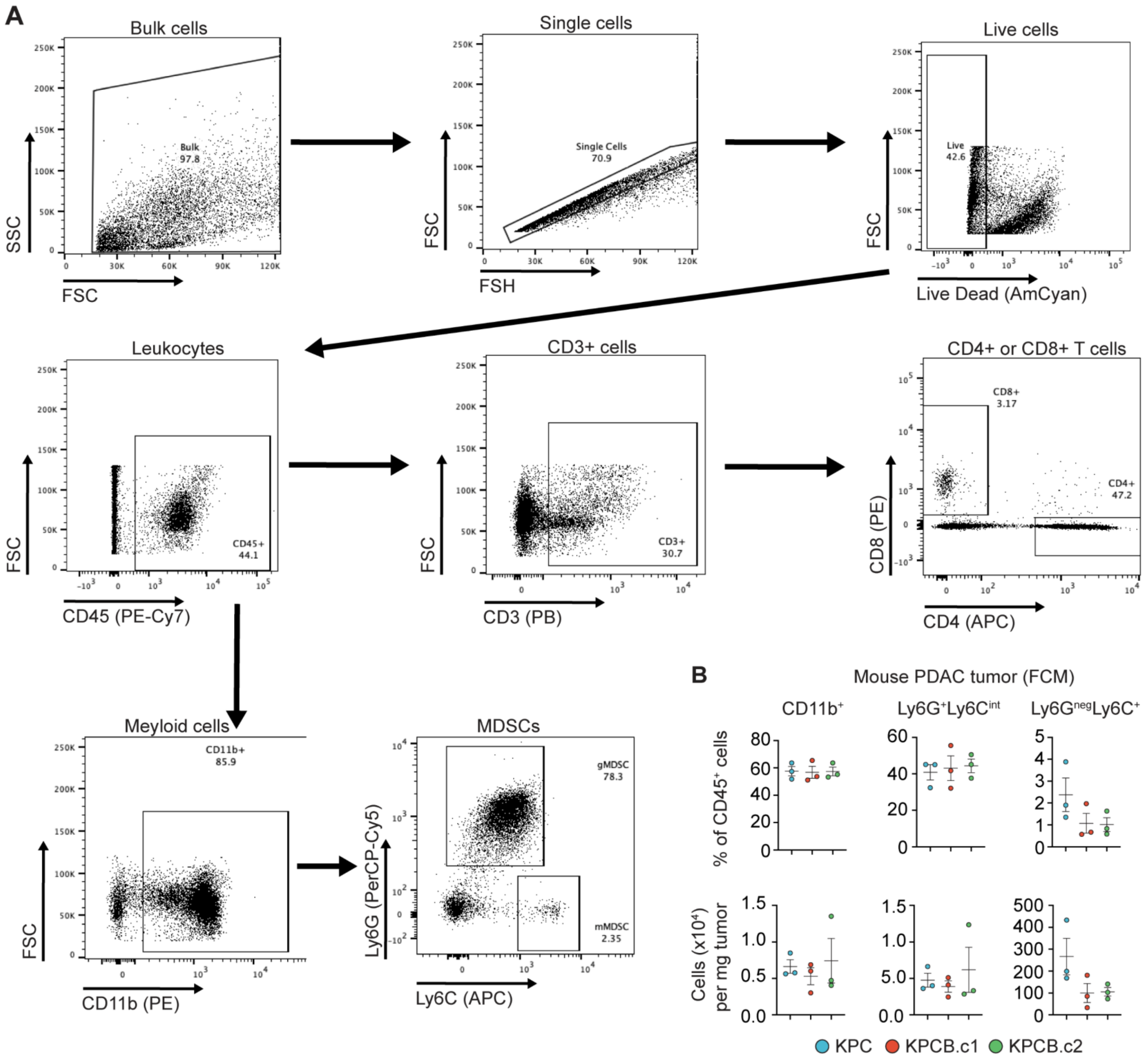

Supplemental Figure 2. Characterization of *Brca2* mutated pancreatic cancer tumor microenvironment. A) Flow gating strategy for characterizing T cell and myeloid cell populations. **B)** Flow cytometry analysis of myeloid cell populations from *Brca2* wild type and *Brca2* mutated pancreatic cancer mouse models. One way ANOVA with Tukey correction for multiple comparisons was used to determine statistical significance. Statistical significance denoted as *, p>0.05; **, p<0.01; ***, p<0.001; and ****, p<0.0001.

Supplemental Figure 3

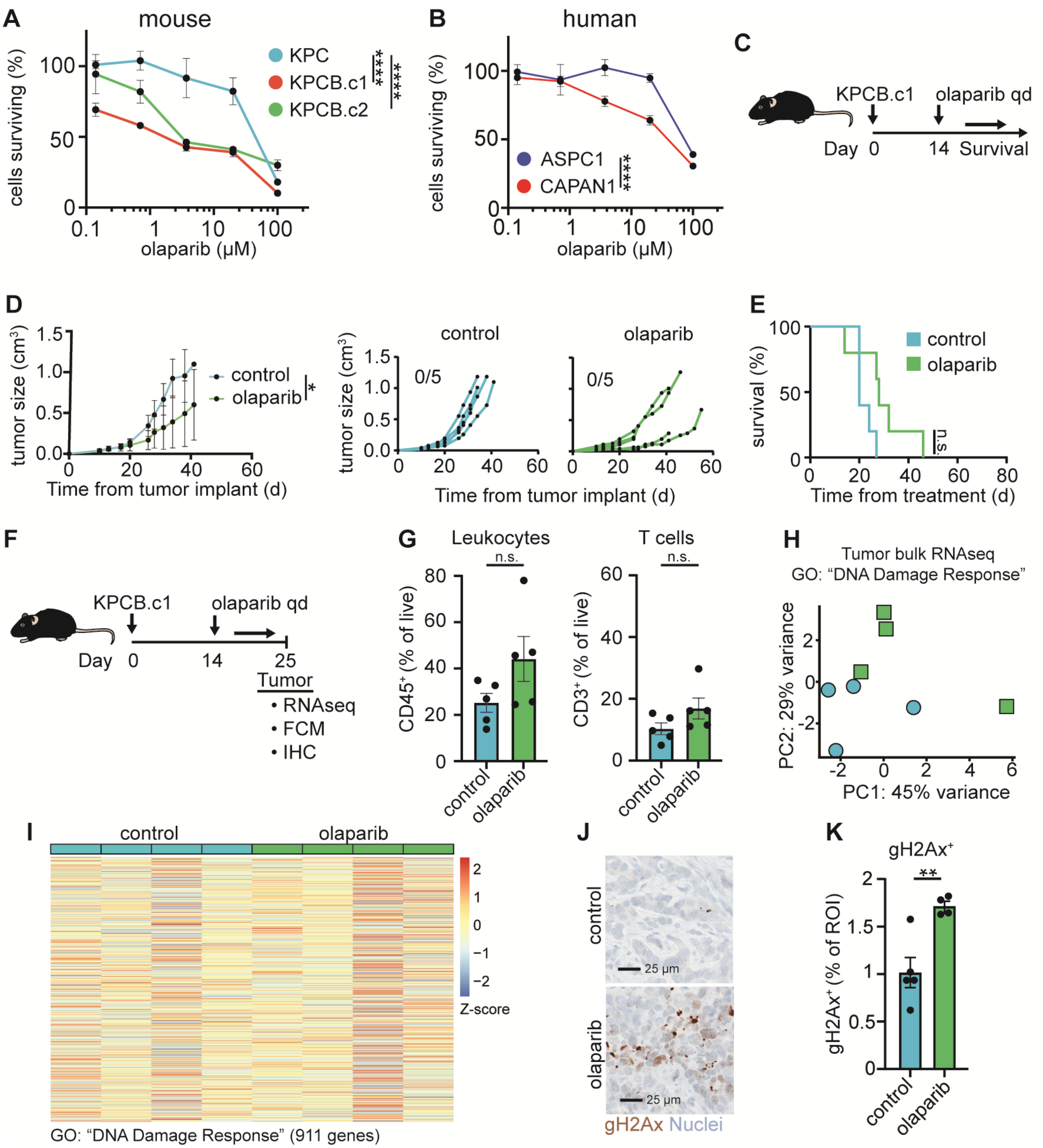

Supplemental Figure 3. Limited efficacy of PARP inhibition monotherapy in *Brca2* mutated pancreatic cancer mouse model. A) MTT assay of KPC, KPCB.c1 or KPCB.c2 at varying doses of olaparib. **B)** MTT assay of ASPC1 (human PDAC non-BRCA mutated) and CAPAN1 (human PDAC BRCA2 mutated) cell lines at varying doses of olaparib. **C)** Experimental design for D-E. **D)** Tumor growth curves. Numbers represent survival at end of experiment. **E)** Kaplan-Meier plot. **F)** Experimental design for G-K. **G)** Flow cytometry analysis of leukocytes and T cells. **H)** Principal component analysis of GO: DNA Damage Response genes from bulk RNA sequencing. **I)** Heatmap showing all 911 gene in the GO: DNA Damage Response gene set in tumors. **J)** Representative IHC images showing γH2Ax expression in tumors. **K)** Quantification of J. Two-way ANOVE, Log-rank test or Mann-Whitney test were used to determine statistical significance. Statistical significance denoted as *, p<0.05 *; **, p<0.01; ***, p<0.001; and ****, p<0.0001.

Supplemental Figure 4

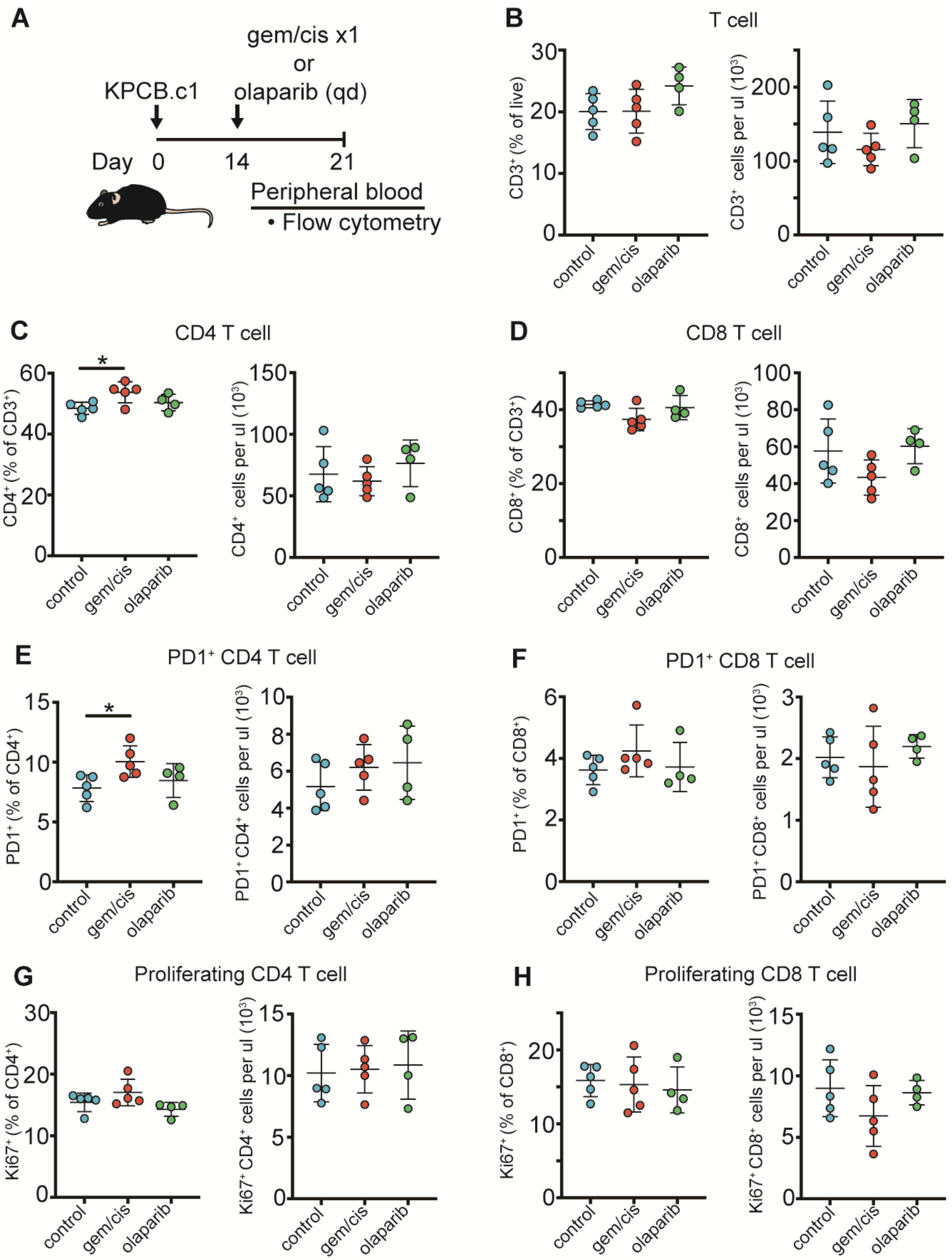

Supplemental Figure 4. Impact of induction chemotherapy and olaparib on systemic T cells. A) Experimental design for B-H. **B)** Flow cytometry analysis of peripheral blood to detect **B)** CD3+ T cells, **C)** CD4+ T cells, **D)** CD8+ T cells, **E)** PD1+ CD4+ T cells, **F)** PD1+ CD8+ T cells, **G)** proliferating Ki67+ CD4+ T cells, and **H)** proliferating Ki67+ CD8+ T cells. One way ANOVA with Tukey correction for multiple comparisons was used to determine statistical significance. Statistical significance denoted as *, $p<0.05$; **, $p<0.01$; ***, $p<0.001$; and ****, $p<0.0001$.

Supplemental Figure 5

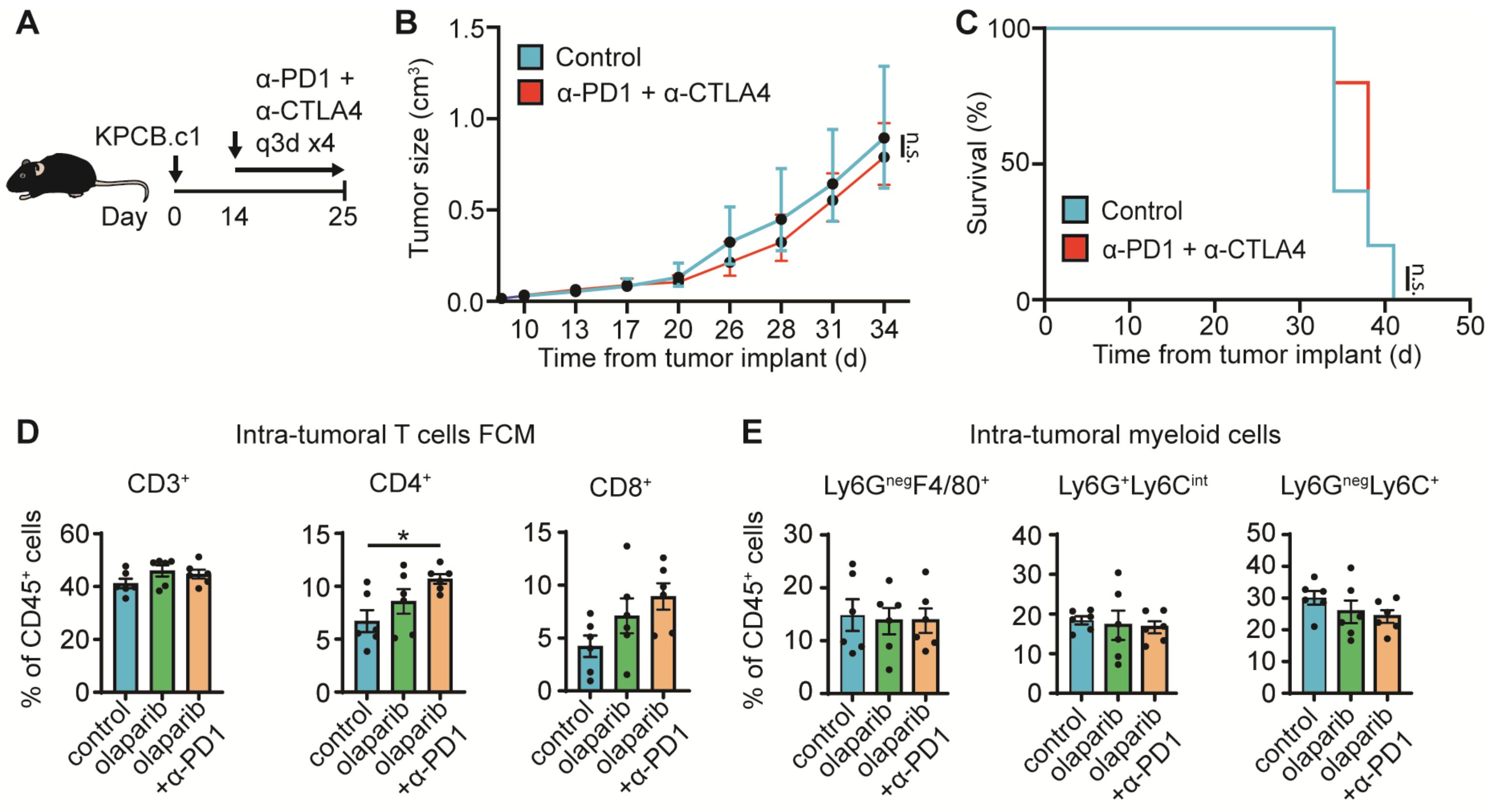

Supplemental Figure 5. *Brca2* mutated murine PDAC is resistant to dual immune checkpoint blockade with anti-PD1/CTLA4. A) Experimental design for B-C. **B)** Tumor growth curves. **C)** Kaplan-Meier plot. **D)** Flow cytometry analysis of intratumor leukocytes, T cells, CD4+ T cells and CD8+ T cells from experiment described in Figure 5a. **E)** Flow cytometry analysis of intratumor myeloid cell populations from experiment in Figure 5a. Mann-Whitney test, one way ANOVA with Tukey correction for multiple comparisons and Log-rank test were used to determine statistical significance. Statistical significance denoted as *, p>0.05; **, p<0.01; ***, p<0.001; and ****, p<0.0001.

Supplemental Figure 6

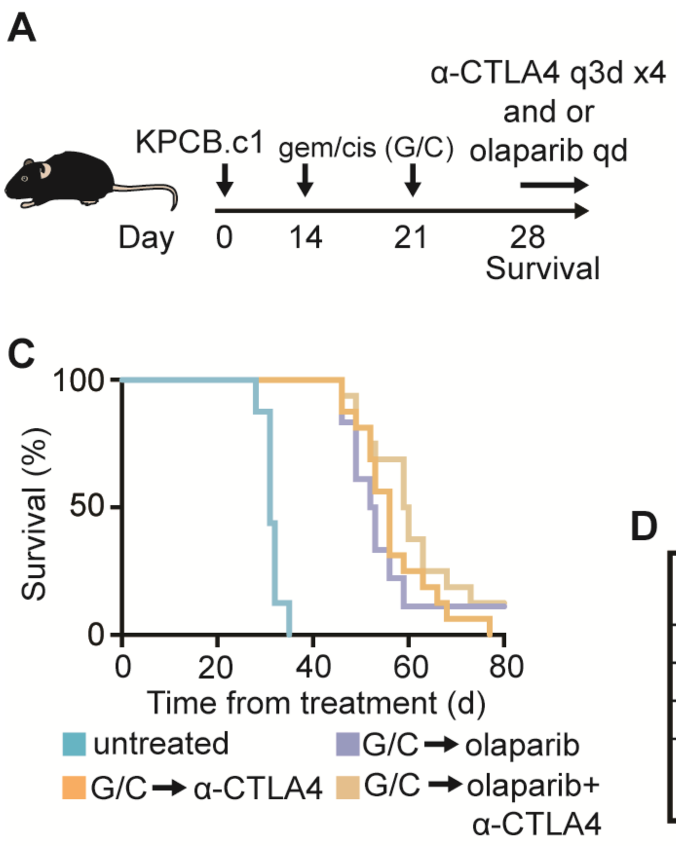

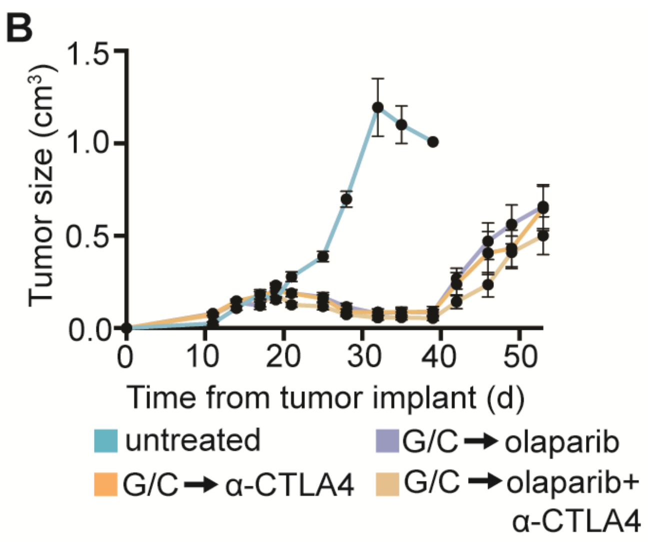

D

	Median survival (d)	Survival
untreated	31	0.0%
G/C → α-CTLA4	56	0.0%
G/C → olaparib	52	11.1%
G/C → olaparib+ α-CTLA4	59	11.1%

Supplemental Figure 6. Anti-CTLA4 therapy does not improve olaparib maintenance strategy in a *Brca2* mutated mouse model. A) Experimental design for B-D. **B)** Tumor growth curves. **C)** Kaplan-Meier plot.. **D)** Table showing median survival and percent survival at the end of study. Data provided is until 95 days from tumor implantation. Two-away ANOVA and Log-rank tests were used to determine statistical significance. Statistical significance denoted as *, p>0.05; **, p<0.01; ***, p<0.001; and ****, p<0.0001.

Supplemental Table 1

**Table S1. Demographic and Characteristics - resection specimen cohort.**		
**Characteristic**		***n* = 8**
Age at diagnosis - yr		
Mean		61
Range		44 - 68
Sex - no. (%)		
Male		3 (37)
Female		5 (63)
Race or ethnic group - no. (%)		
White		4 (50)
Black		1 (13)
Not reported		3 (37)
Neo-adjuvant platinum chemotherapy - no. (%)		
Yes		3 (37)
	Oxaliplatin	2
	Carboplatin	1
No		5 (63)
Stage at diagnosis - no. (%)		
IA		1 (13)
IIA		0 (0)
IIB		7 (87)
Germline mutation - no. (%)		
BRCA1#		1 (13)
BRCA2		7 (87)
PALB2		1 (13)

#One patient identified to have germline mutations in *BRCA1* and *BRCA2*.

Supplemental Table 2

Table S2. Flow cytometry antibodies					
**Antibody**	**Source**	**Product #**	**Tag**	**Clone**	**RRID**
CD8b	BioLegend	126608	PE	YTS156.7.7	AB_961298
CD11b	BD Biosciences	553311	PE	M1/70	AB_394775
CD45	BD Biosciences	550994	PerCP Cy5.5	30-F11	AB_394003
Ly6G	BD Biosciences	560602	PerCP Cy5.5	1A8	AB_1727563
CD3	BioLegend	100220	PE-Cy7	17A2	AB_1732057
CD45	BD Biosciences	552848	PE-Cy7	30-F11	AB_394489
F4/80	eBioscience	17-4801-82	APC	BM8	AB_2784648
CD4	eBioscience	17-0042-82	APC	RM4-5	AB_469323
Ly6C	BioLegend	128016	APC	HK1.4	AB_1732076
Ly6C	BD Biosciences	560596	APC-Cy7	AL-21	AB_1727555
CD3	BioLegend	100214	Pacific Blue	17A2	AB_493645
CD8	BioLegend	100725	Pacific Blue	53-6.7	AB_493426
